# Visible light-driven CdSe nanotube array photocatalyst

**DOI:** 10.1186/1556-276X-8-230

**Published:** 2013-05-16

**Authors:** Haojun Zhu, Quan Li

**Affiliations:** 1 Department of Physics, The Chinese University of Hong Kong, Shatin, New Territories, Hong Kong

**Keywords:** Nanotube arrays, Template synthesis, II-VI semiconductors, Visible light photocatalysis

## Abstract

Large-scale CdSe nanotube arrays on indium tin oxide (ITO) glass have been synthesized using ZnO nanorod template. The strong visible light absorption in CdSe, its excellent photoresponse, and the large surface area associated with the tubular morphology lead to good visible light-driven photocatalytic capability of these nanotube arrays. Compared to freestanding nanoparticles, such one-piece nanotube arrays on ITO make it very convenient for catalyst recycling after their usage

## Background

The development of nanometer-sized photocatalysts for efficient degradation of organic pollutants has attracted continuous research attention [1-4]. Among various morphologies of nanostructures, well-aligned pseudo-one-dimensional (1D) nanostructures such as nanowire (NW) or nanotube (NT) arrays are of particular interest, since the specific morphology brings in several advantages: Its large surface-to-volume ratio prompts the surface-related chemical reactions, which is critical in most of the catalytic processes; when organized into arrays, the ordered NW/NT provides a direct pathway for charge carrier transfer to the conductive substrate. In addition, the NW/NT arrays may enhance light absorption by reducing the reflection or extending the optical path in the nanostructures [5,6].

The most extensively studied NW/NT array photocatalyst for photodegradation of organic pollutants is the titanium dioxide (TiO_2_) nanotube arrays, as it is environmentally benign, capable of total mineralization of organic contaminants, easy to fabricate, and cheap. Nevertheless, its large bandgap (3.2 eV for anatase and 3.0 eV for rutile) only allows the absorption in UV range of the solar spectrum. Although doping TiO_2_ with elements, such as V, Cr, Mn, Fe, C, N, S, F, etc., could extend the absorption spectrum of TiO_2_ to the visible region, other problems occur and lead to the decrease in the quantum efficiency [7,8]. Alternatively, direct employment of the narrower bandgap materials as the photocatalyst has been proposed as a possible solution. A few semiconductors have been investigated, such as II-VI materials (e.g., CdS [2,9] and CdSe [10,11]) and transition metal oxides (e.g., WO_3_[12-14], Fe_2_O_3_[15-18], Cu_2_O [19], Bi_2_WO_6_[20,21], and ZnFe_2_O_4_[22]). Nevertheless, most of the photocatalysts developed are the nanoparticles, which would not enjoy the advantage of the 1D morphology. In addition, after the nanoparticles are dispersed in the waste water for the catalytic reactions, it is troublesome to collect them after use.

In the present work, well-aligned CdSe nanotube arrays on indium tin oxide (ITO)/glass are obtained by electrodepositing CdSe on the surface of ZnO nanorod followed by ZnO etching. Such nanotube arrays exhibit strong light absorption and high photocurrent in response to the visible light. Moreover, the nanotube arrays exhibit good visible light-driven photocatalytic performance, as revealed by the photodegradation of methylene blue (MB) in aqueous solution. The charge carrier flow during the degradation process and mechanism of MB degradation are also discussed.

## Methods

The CdSe nanotube arrays were synthesized via a ZnO nanorod template method, the detail of which can be found elsewhere [23-25]. Briefly, ZnO nanorod arrays were first fabricated on ITO/glass (10 Ω/□) using the hydrothermal method [26-29]. Next, CdSe nanoshells were electrodeposited on the surface of ZnO nanorods from an aqueous solution galvanostatically (at approximately 1 mA/cm^2^) at room temperature in a two-electrode electrochemical cell, with the nanorod array on ITO as the cathode and Pt foil as the anode. The deposition electrolyte contains 0.05 M Cd(CH_3_COO)_2_, 0.1 M Na_3_NTA (nitrilotriacetic acid trisodium salt), and 0.05 M Na_2_SeSO_3_ with excess sulfite [30,31]. After approximately 7 min of electrodeposition, the ZnO/CdSe nanocable arrays were dipped into a 25% ammonia solution at room temperature for 30 min to remove the ZnO core - a process that leads to the formation of nanotube arrays on ITO. Finally, the nanotube samples were annealed at 350°C under Ar atmosphere for 1 h.

The general morphology and the crystallinity of the samples were examined by scanning electron microscopy (SEM; Quantum F400, FEI Company, Hillsboro, USA) and X-ray diffraction (XRD; Rigaku SMARTLAB XRD, Tokyo, Japan), respectively. Their detailed microstructure and chemical composition were investigated using transmission electron microscopy (TEM; Tecnai 20 FEG, FEI Company) with an energy-dispersive X-ray (EDX) spectrometer attached to the same microscope. Optical absorption was measured using a Hitachi U3501 spectrophotometer (Hitachi, Tokyo, Japan). Photoelectrochemical measurements were carried out in a three-electrode electrochemical cell using an electrochemical workstation (CHI660C, Shanghai Chenhua Instruments Co., Ltd., Shanghai, China) with 0.35 M Na_2_SO_3_ and 0.24 M Na_2_S solution as the hole scavenger electrolyte, CdSe nanotube arrays on ITO as the working electrode, Ag/AgCl as the reference electrode, and Pt foil as the counter electrode. The illumination source was the visible light irradiation (100 mW/cm^2^) from a 150-W xenon lamp (Bentham IL7, Berkshire, UK) equipped with a 400-nm longpass filter. Photocatalytic activities of the nanotube arrays were evaluated from the degradation of 0.5 ppm MB aqueous solution (5 ml) with and without adding 10 vol.% ethanol. The degradation process was monitored by measuring the absorbance of the MB solution at 664 nm using Hitachi U3501 spectrophotometer every 0.5 h.

## Results and discussion

### Morphology, crystal structure, and chemical composition

**Figure 1:**
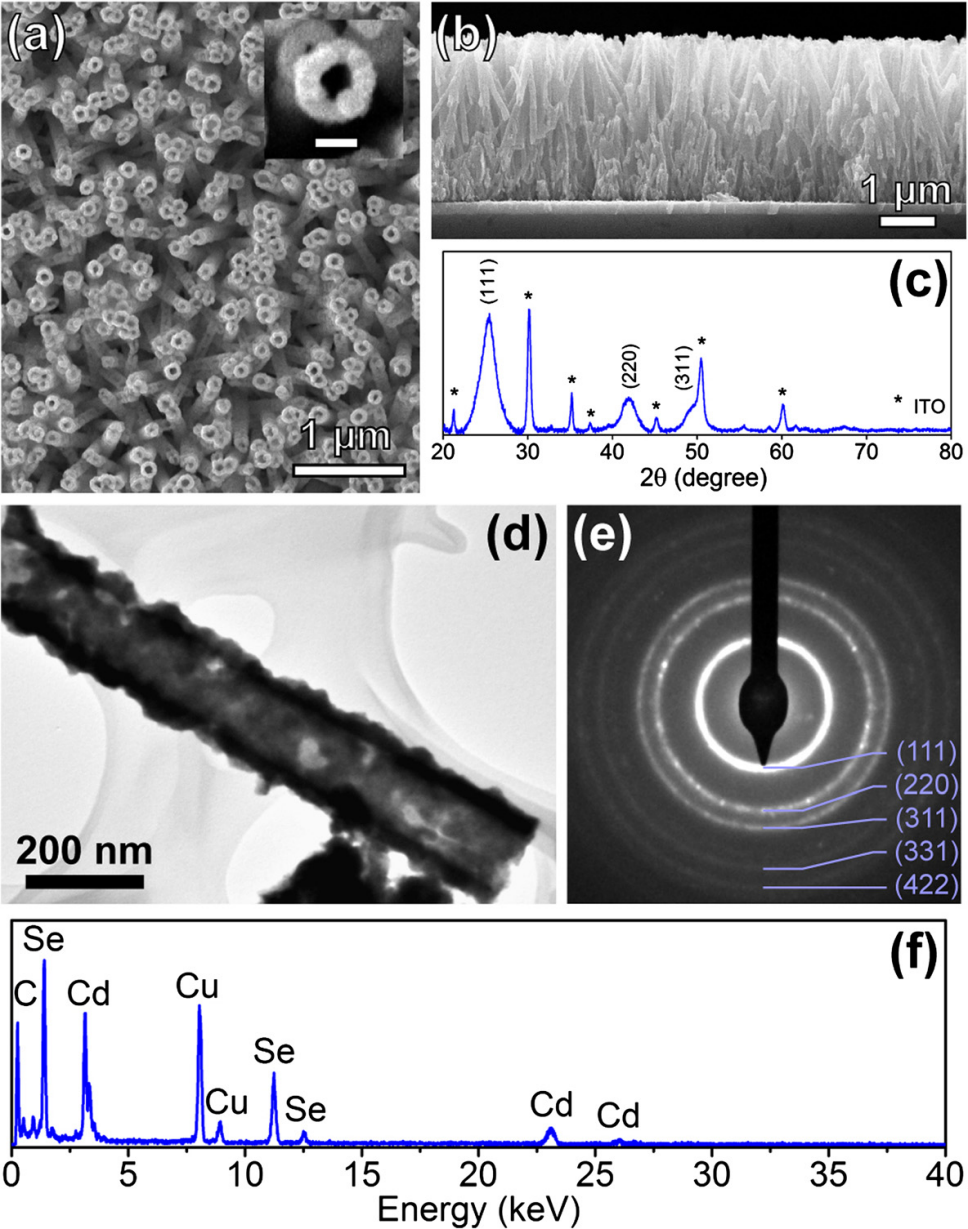
**Morphology, crystal structure, and chemical composition.** (**a**) Top-view and (**b**) side-view SEM images of the typical CdSe nanotube arrays on ITO/glass; the inset in (a) shows the magnified SEM image of a single nanotube (scale bar, 100 nm). (**c**) The XRD data of the sample (the diffraction peaks from the ITO substrate are marked with asterisks). (**d**) The TEM image, (**e**) the SAD pattern, and (**f**) the EDX spectrum taken from a single CdSe nanotube.

Figure 1a,b shows top-view and side-view SEM images of typical CdSe nanotube arrays. The inner diameters, wall thicknesses, and lengths of the nanotubes are estimated as approximately 70 nm, approximately 50 nm, and approximately 2.5 μm, respectively. The inner diameters and the lengths of the nanotubes are inherited from the original ZnO nanorod template, the size of which is tunable. The wall thickness of the CdSe nanotube can be varied by adjusting the electrochemical deposition time. Detailed discussion on the nanotube morphology control can be found in previous works [23]. XRD pattern taken from the annealed nanotube array sample is shown in Figure 1c, in which the diffraction peaks from the ITO substrate are marked with asterisks. All remaining peaks can be assigned to the cubic zinc blende (ZB) structure of CdSe (JCPDS no. 88-2346). ZnO diffraction has not been detected, suggesting that most of the ZnO cores have been removed by the ammonia etching. The full width at half maximum of the CdSe diffraction peaks is rather large, suggesting the small grain size in the sample. The crystalline size is estimated to be around 5 nm by Scherrer's equation [32,33]. Distinct tubular structure can also be seen in the TEM image (Figure 1d) taken from the same sample, and the polycrystalline nature of the nanotube is suggested by the patch-like contrast along the tube wall. Selected area diffraction (SAD), taken from a single nanotube (Figure 1e) shows a ring pattern that can be indexed to ZB CdSe being consistent with the XRD result. EDX analysis of the nanotube shows that it is composed of Cd and Se only, with Cd to Se ratio approximately equals 1 (Figure 1f; the C and Cu signals in the EDX spectrum come from the TEM grid).

### Optical properties

**Figure 2:**
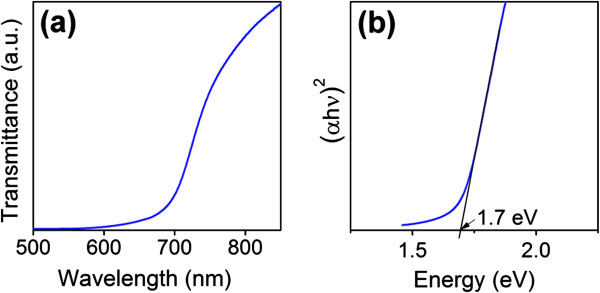
**Optical properties.** (**a**) Optical transmittance spectrum of CdSe nanotube arrays on ITO. (**b**) The corresponding plot of (*α*h*ν*)^2^ vs. h*ν* to determine its optical bandgap.

Figure 2a shows the typical optical transmittance spectra of CdSe nanotube arrays on ITO. Strong visible light absorption is observed with a rather sharp bandgap absorption edge at approximately 700 nm. Estimation of the bandgap of the CdSe nanotube samples has been made from the absorption spectrum (Figure 2b). For direct optical transitions (i.e., CdSe in the present case), the relationship between the absorption coefficient, *α*, and incident photon energy, hν, near the band edge can be expressed as follows:

$$\alpha =\frac{A}{\mathrm{h\nu}}{\left(\mathrm{h\nu}-E_{g}\right)}^{\frac{1}{2}},$$

where *A* is a constant and *E*_g_ is the optical bandgap. By plotting (*α*hν)^2^ as a function of hν, one can determine *E*_g_ by extrapolating the linear portion of the curve to intersect energy axis [34,35]. The optical bandgap of CdSe nanotube arrays is determined as approximately 1.7 eV being consistent with the literature value of CdSe [36].

### Photoelectrochemical performance

**Figure 3:**
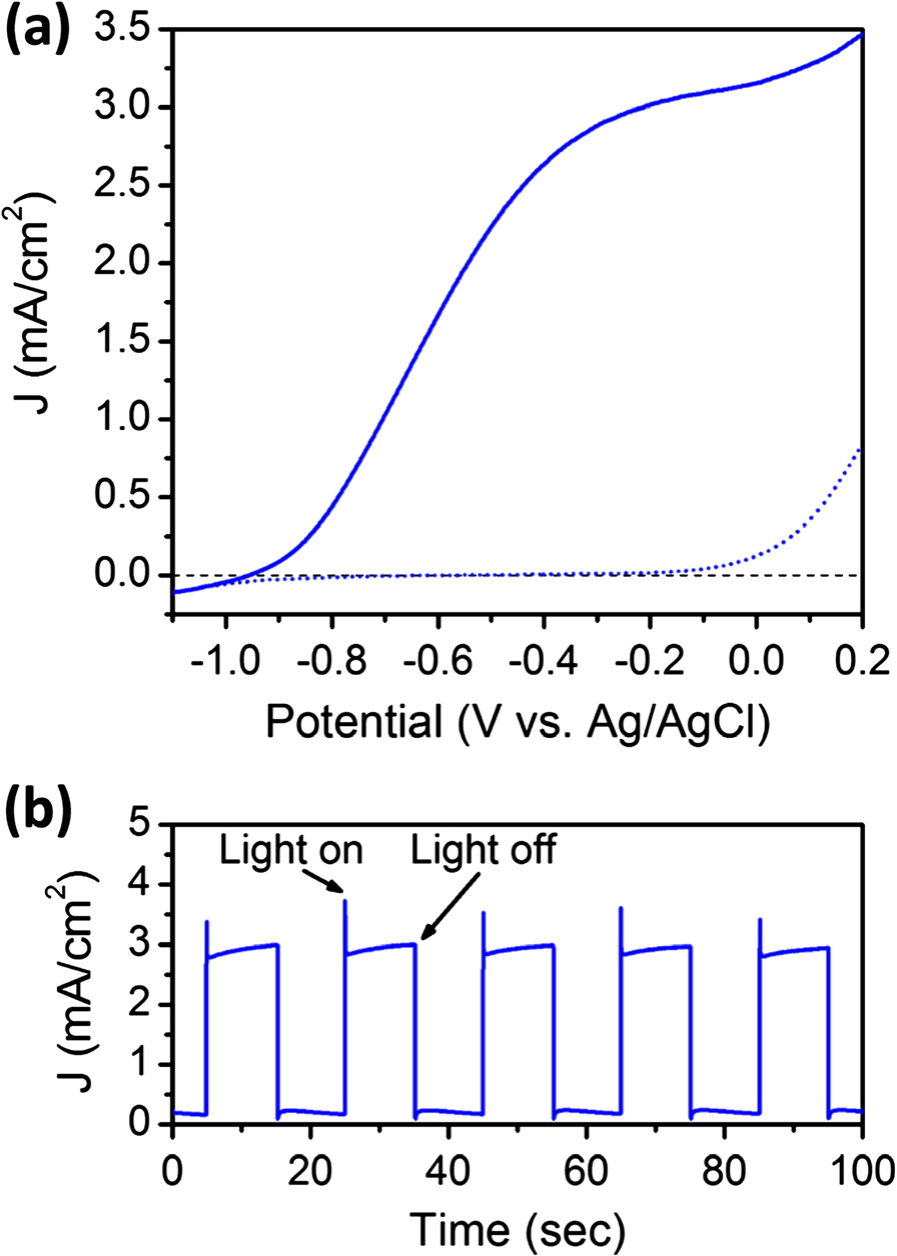
**PEC performance.** (**a**) Current density-potential (*J*-*V*) characteristics obtained from CdSe nanotube arrays under dark conditions and visible light illumination (*λ* > 400 nm, 100 mW/cm^2^). The scan rate is 10 mV/s. (**b**) The photocurrent response to on-off cycles of illumination at a constant potential of −0.2 V vs. Ag/AgCl.

The photoelectrochemical measurements were performed under visible light illumination (*λ* > 400 nm, 100 mW/cm^2^) in the sulfide-sulfite (S^2−^/SO_3_^2−^) aqueous electrolyte to suppress the photocorrosion of CdSe nanotubes [37-41]. The photoelectrochemical (PEC) performance of CdSe nanotube arrays under dark and illumination conditions are presented in Figure 3a. In the dark, the current density-potential (*J*-*V*) characteristics shows a typical rectifying behavior, with a small current density of 1.8 × 10^−2^ mA/cm^2^ at a potential of −0.2 V (vs. Ag/AgCl). When the photoelectrode is illuminated by the visible light, the photocurrent density shows a two orders of magnitude increase to 3.0 mA/cm^2^ at the same potential. The positive photocurrent indicates that CdSe nanotubes act as photoanode being consistent with the n-type conductivity of unintentionally doped CdSe. During repeated on-off cycles of illumination (Figure 3b), prompt and steady photocurrent generation can be obtained, which indicates the fast photoresponse of CdSe nanotube arrays and neglectable photocorrosion to the electrode. These results are comparable to the best PEC performance of similar type of photoelectrodes in the literatures [40,41].

### Photocatalytic activities

**Figure 4:**
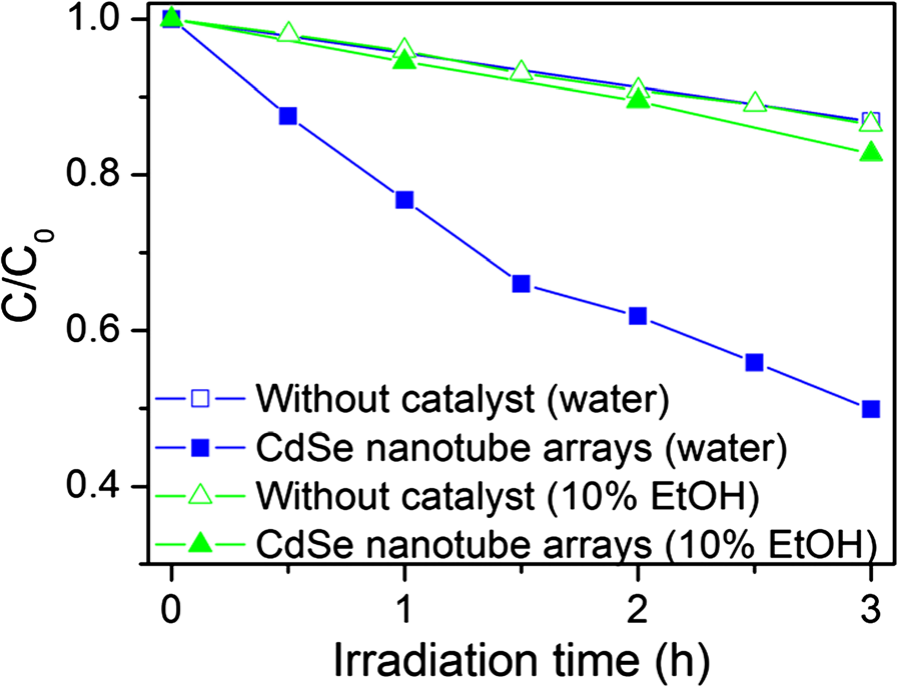
**Photocatalytic degradation performance.** Photocatalytic degradation performance of CdSe nanotube arrays on ITO under visible light irradiation (*λ* > 400 nm) in the MB aqueous solution (blue symbols) and the solution added with 10 vol.% ethanol (green symbols). *C* is the concentration of MB at a given reaction time; *C*_0_ is the initial concentration of MB.

In order to evaluate the photocatalytic performance of CdSe nanotube arrays on ITO, the degradation of MB was chosen as a probe for photoreaction. The results indicate that CdSe nanotubes were efficient in the photodegradation of MB under visible light irradiation (blue symbols in Figure 4). The degradation reaction of MB can be described as a pseudo-first-order reaction with the kinetics expressed by the following equation when the MB concentration is low (<1 mM):

$$\ln\frac{C_{0}}{C}=\mathrm{kt},$$

where *C*_0_ is the initial concentration of MB in the solution; *C*, the concentration of MB at a given reaction time, *t*; and *k*, the reaction rate constant [42]. From the linear extrapolations, the calculated reaction rate constant of the nanotube arrays is estimated to be 3.3 × 10^−3^ min^−1^ after subtracting the direct photolysis of MB. The cycling properties of CdSe nanotube arrays were also studied. The photocatalyst shows a slight decrease in the catalytic activities after being tested for three times (Additional file 1: Figure S1).

**Figure 5:**
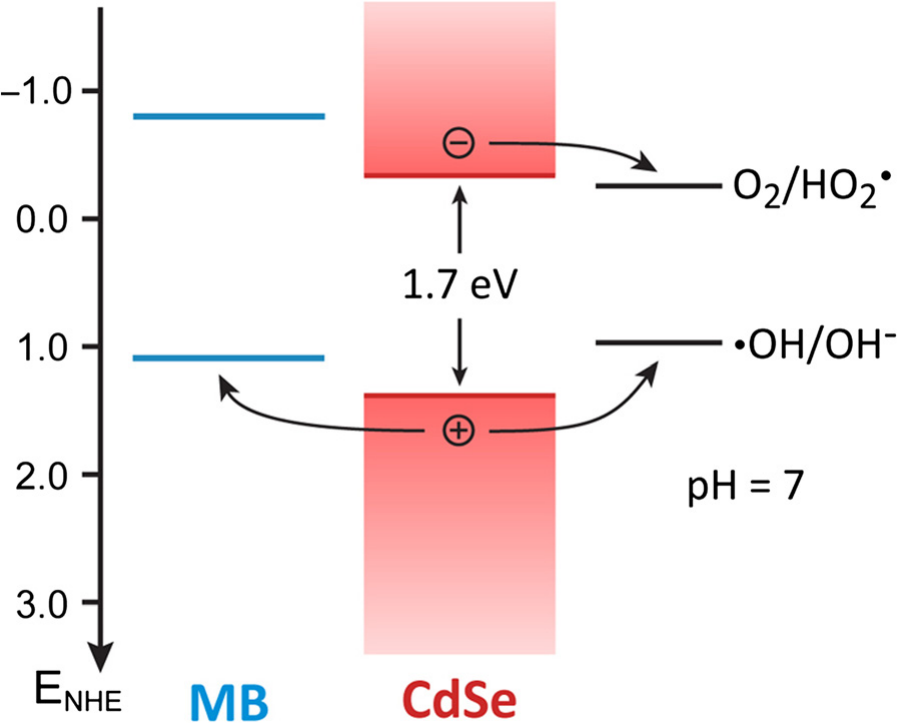
Schematic showing the mechanism of MB photodegradation by CdSe nanotube arrays.

The photocatalytic degradation mechanism of CdSe nanotube arrays is proposed in Figure 5. The energy diagram shows that the valence band maximum (VBM) of CdSe is more positive than the oxidation potential of MB and the redox potential *E*(**·**OH/OH^−^). The conduction band minimum is more positive than the reduction potential of MB but negative than the redox potential *E*(O_2_/HO_2_**·**) [43-45]. Upon visible light irradiation, electron-hole pairs are generated (Equation 1) in the CdSe, and their separation is driven by the band bending formed at the interface of CdSe and the solution. The n-type conductivity of unintentionally doped CdSe promotes the charge carrier separation. The photogenerated holes oxidize MB molecules directly (Equation 2) and/or hydroxide ion (OH^−^) to produce **·**OH radicals (Equation 3), which also contribute to MB degradation via other route (Equation 4). At the same time, the photogenerated electrons can reduce the oxygen adsorbed on the catalyst (Equation 5), resulting in free HO_2_**·** radicals, which also contribute to the oxidation of MB. However, such electron injection is not efficient due to the small offset between the VBM of CdSe and *E*(O_2_/HO_2_).

(1) $$\mathrm{CdSe}+h\nu \to h^{+}+e^{–}$$

(2) $$h^{+}+\mathrm{MB}\to \mathrm{degradation}\;\mathrm{products}$$

(3) $$h^{+}+{\mathrm{OH}}^{–}\to \cdot \mathrm{OH}$$

(4) ·OH+MB→degradationproducts

(5) $$e^{–}+O_{2}+H_{2}O\to {\mathrm{HO}}_{2}\cdot +{\mathrm{OH}}^{–}$$

Further evidence for the proposed photodegradation mechanism is obtained by adding ethanol (10 vol.%) to the MB aqueous solution. This alcohol has been found to scavenge both holes and **·**OH radicals [46]. As a result, MB degradation is completely quenched after adding ethanol (green symbols in Figure 4), supporting that the photogenerated holes and/or **·**OH radicals are mainly responsible for the MB degradation.

## Conclusions

In conclusion, large-scale CdSe nanotube arrays on ITO have been obtained by electrodepositing CdSe on the surface of ZnO nanorods followed by ZnO etching. The nanotube arrays show a strong absorption edge at approximately 700 nm, high photoresponse under visible light illumination, and good visible light-driven photocatalytic capability. This nanotube array on substrate morphology provides a device like catalyst assembly without sacrificing the surface area and is very attractive due to the recycling convenience after usage, as compared to freestanding nanostructures.

## Competing interests

The authors declare that they have no competing interests.

## Authors’ contributions

HZ carried out the sample preparation, performed the sample characterization, and wrote the paper. QL supervised the whole work and revised the manuscript. All authors read and approved the final manuscript.

## Supplementary Material

Additional file 1: Figure S1 Cyclic photodegradation of MB by the CdSe nanotube arrays for three times. Click here for file
