# Supplementary material for: Visible light-driven CdSe nanotube array photocatalyst
Source: Nanoscale Res Lett. 2013 May 16;8(1):230. doi: 10.1186/1556-276X-8-230 (PMC3663676; doi:10.1186/1556-276X-8-230)
Supplement: Additional file 1: Figure S1 — Cyclic photodegradation of MB by the CdSe nanotube arrays for three times. [file 1556-276X-8-230-S1.docx]

**Figure S1.** Cyclic photodegradation of MB by the CdSe nanotube arrays for three times.
